# The critical role of primary care health care professionals in referring older adults to community-based fall prevention programs

**DOI:** 10.3389/fpubh.2024.1377972

**Published:** 2024-03-13

**Authors:** Jonathan Howland, Elizabeth W. Peterson

**Affiliations:** ^1^Department of Emergency Medicine, Boston University Chobanian and Avedisian School of Medicine, Boston, MA, United States; ^2^Department of Occupational Therapy, University of Illinois Chicago, Chicago, IL, United States

**Keywords:** older adults, falls, primary care, falls prevention, community-based programs

## Introduction

Among older adults, falls are common and the leading cause of fatal and non-fatal injuries (1). In the United States, one in four older adults ages 65 and older reports falling each year (2). On average, 100 older adults died every day because of falls in 2021 (2) and estimates of medical costs of fatal and non-fatal older adult falls are ~$50 billion annually (3). About 20% of falls require medical attention (4). Falls leading to injuries can affect levels of activity, psychosocial status, and quality of life. Even when falls do not require medical attention, the experience of falling can result in fear of falling (5). While a reasonable level of concern can prevent engagement in risky activities, fear of falling that is disproportionate with functional abilities can prevent engagement in activities necessary to maintain health and wellbeing. Fear of falling is associated with depressive symptomatology (6), impacts gait (7), leads to activity curtailment (8–10), and increased fall risk (5, 8).

## Trends

Between 2001 and 2021, the number of Americans dying from unintentional falls increased from 15,000 to over 44,000 as the crude death rate rose from 5.3 (per 100,000 population) to 13.5 (1). No demographic is unaffected. Among racial and ethnic groups, White older adults have the highest death rate from falls, and the biggest increase, however death rates are rising among Black seniors, Hispanic seniors, Asian seniors, and Native American seniors alike (11). While this trend is not completely understood, there may be several contributors. Some of the increase in fall mortality may be due to innovations in medical record keeping that document causes and circumstances of injuries (12), demographic trends and pharmacological factors are also at play. Older adults are living longer. Globally, the number of persons aged 60 years or over is expected to more than double, from 841 million people in 2013 to more than 2 billion in 2050 (13) and many are or will be living with frailty, co-morbidities and chronic health conditions that could increase fall risk (14, 15). Fourteen medication classes, most of which are psychotropic medications, have been identified as fall-risk increasing drugs (FRID) (16). Although falls are widely recognized as common and preventable adverse drug events (17), healthcare professionals must make decisions about deprescribing FRID that consider patient preferences and the trade-offs between competing health conditions (18, 19).

### Community-based fall prevention interventions

The value of multifactorial, individualized clinical interventions in preventing falls among community-dwelling older adults at risk for falls is well-established (20). For older adults at low risk for falls, primary prevention involving annual re-assessment of fall risk is recommended (20). Community-based fall prevention programs are important compliments to clinical approaches to fall risk management and several have been evaluated through randomized trials and found to reduce fall risk. These programs reflect diverse approaches, ranging from exercise-based programs (e.g., *Tai Ji Quan: Moving for Better Balance* or *Enhanced Fitness*, an arthritis friendly falls prevention program) to interventions featuring cognitive behavioral therapy to address activity restriction associated with fear of falling (e.g., *Matter of Balance* (MOB) (21). Importantly, these programs are cost effective. Carande-Kulis et al. (22) estimated the net benefit and return on investment (ROI) of three evidence-based fall prevention programs. *Otago*, a program that targets frail older adults and is delivered at home by a physical therapist or physical therapist assistant, had a 1-year net benefit of $121.85 and 36% ROI. *Tai Chi: Moving for Better Balance*, a group program for enhancing strength and balance, had a 1-year net benefit of $529.86 and 509% ROI. *Stepping On*, a program focusing on balance and strength exercises; medication review; vision review; and home modifications, had a 1-year net benefit of $134.37 and 64% ROI (22).

Ackerman et al. found that *Enhanced Fitness* participants had similar total healthcare costs to non-participants during year 1 of the program, but during Year 2, participants' adjusted total healthcare costs were $1,186 lower (*p* < 0.005) healthcare costs of non-participants (23). In a separate study, Howland et al. investigated the ROI on MOB and estimated a ROI of 144% if all older adults presenting at Massachusetts Emergency Departments were referred to MOB and 50% of the older adults referred complied by enrolling in the program (24). The Centers for Medicare and Medicaid Services (CMMS) conducted a retrospective cohort study evaluating the cost impact of MOB and found that compared to controls (matched on prior year healthcare utilization and costs, co-morbidities, demographics, and region of residence) those who had participated in MOB had significantly lower overall healthcare costs ($938) and healthcare utilization, and reduced mortality during the post-participation year (25).

### Primary care referrals to community-based fall prevention programs

Notwithstanding evidence of effectiveness and cost-effectiveness, community-based programs have not had population level effects on older adult falls, in part because referral to these programs has not been well integrated into primary care practice. Smith et al. surveyed health care providers in New York state and reported that < 20% referred older adult patients to community-based fall prevention programs (26). In a survey of primary care providers at two Massachusetts Accountable Care Organizations, Howland et al. (27) found that only 9% reported having referred an older adult patient to a community-based fall prevention program during the previous year. The infrequency of referrals is one aspect of a larger problem: many respondents had limited knowledge of fall risk assessment and intervention. While most respondents believed that older adults should be assessed for fall risk and most believed that assessment would identify risk factors that could be modified, only about half believed that they had the expertise to conduct fall risk assessment and only about two thirds believed that assessing older adult patients for fall risk was prevailing practice among their peers. Only 14% were aware of the *Stopping Elderly Accidents, Deaths and Injuries* (STEADI) toolkit which was created by Centers for Disease Control and Prevention (CDC) to help health care providers incorporate fall risk assessment, treatment, and referral into clinical practice, and to facilitate patient referrals to community-based fall prevention programs (28). Only 15% were familiar with MOB, the most widely disseminated community fall prevention program in Massachusetts (29).

### Self-selection into community-based fall prevention programs is problematic

Community-based fall prevention programs are most often offered by public organizations (e.g., area agencies on aging, township-based park districts) and private organizations (e.g., YMCA's) that serve older adults. While some of these entities may employ health care professionals, most community fall prevention programs are marketed directly to the public. Few participants are referred by healthcare professionals.

This model is problematic. First, fear of falling is an independent fall risk factor (5). Those who self-select for fall prevention programs, by definition, have some measure of fall prevention self-efficacy, while some of those who do not elect to participate may lack falls self-efficacy. Thus, without encouragement from their healthcare professionals, those who may benefit the most from fall prevention programs may be least likely to participate. Second, falls can be a sign of early or underlying health conditions (30), they are reliable and strong predictors of subsequent falls (31, 32) and injurious falls predict future falls requiring medical care (33, 34). Consequently, individualized assessment and targeted intervention is indicated for older adults presenting in an emergency department or other clinical settings with a fall (35, 36). Older adults who self-select into community-based fall prevention programs without interfacing with health care professionals may experience delays in receiving timely medical attention may, or such attention may not be received at all. This compounds a well-documented existing problem: follow-up studies of patients discharged from treatment for fall injury indicate that few are referred to or receive fall-prevention intervention (37–40). Third, to realize their full potential for population-level impact, community-based programs must be broadly disseminated and engage a substantial portion of the population. It is unlikely that community-based fall prevention will be scaled up to yield population-level decreases in older adult fall risk without referrals from health care professionals.

## Discussion

Primary care-based health care professionals are uniquely positioned to refer older adults to evidence-based fall prevention programs and thereby enhance the potential of population level impacts of these and future community-based interventions. In addition to access to older adults presenting at all points on the fall risk continuum, the increase in use of team-based primary care models supports assessment and management of fall risk among older adults. Most falls in community-dwelling older adults result from a combination of risk factors, a multifactorial assessment is indicated for older adults at high risk for falling (20). Interprofessional team members can draw from their collective expertise to work with older adults to identify and prioritize modifiable risk factors and refer older adult patients to community-based programs that best meet individualized needs.

Fortunately, primary care-based health care professionals have several mechanisms available for reimbursement of fall prevention services that can lead to referral to community-based programs. Falls-related services may be reimbursable through negotiation with private plans or by Medicare (41). Annual Wellness Visits (AWV), which the US CMMS began reimbursing in 2011, are a particularly important vehicle for reimbursement. Findings from studies of Texas Medicare data for 2014–2018, highlight the potential for AWV data to inform fall prevention interventions and other health promotion practices (42).

Although health care is shifting away from fee-for-service reimbursement to alternative payment models, fee for service is still the norm for most primary care practices (43). Several Current Procedural Terminology (CPT) codes apply to fall prevention services provided in primary care clinics. For example, occupational therapists working in primary care can apply CPT codes to a range of relevant services, many of which highlight the increasingly evident relationship between self-management of chronic medical conditions and fall prevention (44). These services include but are not limited to individualized strategies for adherence to medications, activity/exercise programs, and education regarding activities of daily living (ADLs), activity modification and general exercise (43).

Finally, primary care providers can utilize resources made available through the National Council on Aging (NCOA) and the CDC. Both the NCOA and CDC are leading efforts to educate healthcare professionals on the importance of referring older adult patients to community-based fall prevention programs. With funding from the Administration on Living (ACL), the NCOA has created the National Falls Prevention Resource Center for Professionals (45). Health care professionals can contact the Center to learn about State Fall Prevention Coalition and local fall prevention programs. The Center also provides a detailed listing of fall prevention programs that meet ACL criteria for evidence-based programs specifically for falls prevention. The aforementioned STEADI Toolkit is a comprehensive set of materials designed for use in primary care that includes the *Algorithm for Fall Risk Screening, Assessment, and Intervention* (28). The algorithm recommends that older adults who are screened, found to be at risk for falls and presenting with poor gait, strength, and balance should be further assessed and also be referred to evidence-based fall prevention programs. The STEADI algorithm also indicates that older adults screened and found not to be at risk for falls should be referred to community exercise or fall prevention programs.

Since STEADI was launched in 2012, important insights into successful implementation of fall prevention practices in primary care have been gained (46). Health care professionals in primary care who combine these lessons learned with strategic efforts to learn about the evidence-based fall prevention programs available in their own health care system or communities will be well-positioned to fulfill the critical role they play in facilitating referrals to community-based fall prevention programs.

## Author contributions

JH: Conceptualization, Writing – original draft, Writing – review & editing. EWP: Conceptualization, Writing – original draft, Writing – review & editing.
